# Ultrasonic microencapsulation of oil-soluble vitamins by hen egg white and green tea for fortification of food

**DOI:** 10.1016/j.foodchem.2021.129432

**Published:** 2021-08-15

**Authors:** Haiyan Zhu, Srinivas Mettu, Francesca Cavalieri, Muthupandian Ashokkumar

**Affiliations:** a School of Chemistry, The University of Melbourne, Parkville, Melbourne, Victoria, 3010, Australia; b Chemical and Environmental Engineering, School of Engineering, RMIT University, Victoria, 3000, Australia; c School of Science, RMIT University, Victoria, 3000, Australia; d Dipartimento di Scienze e Tecnologie Chimiche, Università di Roma “Tor Vergata”, Via Della Ricerca Scientifica 1, 00133, Rome, Italy

**Keywords:** Ultrasonic encapsulation, Oil-soluble vitamins, Egg whites, *In-vitro* digestion, Food fortification

## Abstract

We report the microencapsulation of oil soluble vitamins (A, D and E) using a one pot ultrasonic process and raw egg white proteins as a shell material. Green tea catechin/iron complex coating method was further developed to impart UV filtering property to the microcapsules in order to protect the encapsulated nutrients from photodegradation. The microcapsules showed antibacterial properties and long shelf-life. The encapsulated vitamins were protected from degradation upon heating, UV irradiation, simulated storage/transit and cooking processes. The *in-vitro* digestion study showed that functional vitamin D can be potentially released in the gastrointestinal tract improving vitamin D availability by more than 2-fold compared to the free vitamin. The vitamin D microcapsules were highly stable and maintained their microstructures once incorporated into staple food products. The low-cost egg white shell encapsulated vitamins can improve the nutritional value of staple food products to combat maternal and child malnutrition.

## Highlights

• Highly stable vitamin loaded microcapsules are synthesised by ultrasound.
• Egg white proteins provide robust shells to protect vitamins from degradation.
• External green tea/iron coating imparts UV filtering property to microcapsules.
• Microcapsules maintain structural integrity during food fortification.
• *In-vitro* digestion model shows the effective release of the encapsulated vitamin.

## 1 Introduction

Micronutrients including vitamins and minerals, are essential elements required to assist growth, development, and metabolic processes of human body (Gharibzahedi & Jafari, 2017). Although micronutrients are only required in minimal amounts for orchestrating various physiological functions to maintain health, micronutrients deficiencies affect up to 2 billion people and cause 3 million childhood mortalities each year (Anselmo et al., 2019, Palermo and Holick, 2014, World Health Organization, 2019) primarily in developing countries. Most micronutrients are present in natural food products, but the natural sources of vitamin D are very limited. As an important micronutrient, vitamin D promotes calcium absorption and maintains adequate calcium and magnesium serum concentrations to enable normal mineralization of bone (Thacher & Clarke, 2011). In combination with vitamins A and E, vitamin D exerts antioxidant and anti-inflammatory activities with beneficial effects on immune system. Vitamin D production in the skin by exposure to sunlight is its primary but limited natural source. The deficiencies of vitamin D can be resolved by using food fortification strategies (Akhtar, Ismail, & Hussain, 2019).

Food fortification has been applied in food industry to improve the nutritional value to solve the malnutrition problems (Dary & Hurrell, 2006). In conventional methods, micronutrients being delivered are simply mixed into the bulk. In this case, nutrients are easily exposed to high temperature, oxygen and humidity conditions; hence they are prone to oxidation resulting in the loss of nutritional properties during transit/storage and cooking processes. Compared to the bulk encapsulation methods, microencapsulation has been used for preserving the activity and stability of ingredients such as vitamins and minerals. (Corrêa-Filho et al., 2019, Sagis, 2015, Schrooyen et al., 2001).

A large number of reports in the literature focus on encapsulation of fat-soluble vitamins in microparticles (MPs), nanoparticles, agglomerates, and powders using solvent-evaporation, polymerization, spray-drying and coacervation methods (Anselmo et al., 2019, Li et al., 2011, McClements, 2015, Sagis, 2015, Schrooyen et al., 2001). However, these microencapsulation approaches are limited by the low micronutrients loading and delivery capacity, toxic coating materials and organic solvents, multiple steps for micronutrients encapsulation, and therefore they are unsuitable for food applications. A simple and cost-effective strategy to encapsulate micronutrients into food-grade shells has a great potential to overcome those limitations.

The ultrasound-assisted microencapsulation of ingredients is a straightforward and eco-friendly method which has been widely used to prepare proteinaceous-based microcapsules (Leong, Martin, & Ashokkumar, 2017). Grinstaff and Suslick have first developed an ultrasonic technique to synthesise air or liquid (hexane, cyclohexane, and toluene) filled BSA proteinaceous microspheres (Grinstaff & Suslick, 1991). We extensively contributed to the field of ultrasonic microencapsulation of gases and liquid using lysozyme(Cavalieri et al., 2008, Zhou et al., 2010), pea protein (Ye, Biviano, Mettu, Zhou, Dagastine, & Ashokkumar, 2016) and chitosan (Ye, Mettu, Zhou, Dagastine, & Ashokkumar, 2018). It is noted that microbubbles/capsules synthesized by ultrasound in aqueous media have several advantages over other systems, such as thick and crosslinked shell, controlled size distribution and mechanical strength. Lysozyme based microparticles showed great potential for delivery of therapeutic agents (Lee, Cavalieri, & Ashokkumar, 2019), however the synthesis required an expensive protein isolated from egg white, combined with the use of toxic denaturing agents (DTT). We reasoned that for microencapsulation of micronutrients to be directly employed for fortification of food, raw, edible and non-toxic building blocks available on a large scale would be desirable.

Egg white (EW) proteins are commonly-available and cost-effective food ingredients, which also have high nutritional value. Egg white mainly consists of water and a mixture of proteins, including ovalbumin (54%), ovotransferrin (12%), ovomucoid (11%), lysozyme (3.5%), and ovomucin (3.5%). Compared with other poorly soluble proteins, egg white proteins are water soluble, surface active, therefore EW does not need to be treated by ultrasound to promote its solubility. The high percentage of water-soluble protein in egg white allows it to form a stable foam for cross-linking. Importantly, the antibacterial properties of lysozyme, can potentially confer antibacterial functionality to the microcapsules synthesized via egg white, probably resulting in a longer shelf-life (Cavalieri et al., 2008, Lee et al., 2019).

In the current study, we describe an ultrasonic encapsulation method to form highly stable vitamin D filled microcapsules directly using raw egg white and green tea, without resorting to any pre-chemical/thermal denaturation process. Such ultrasonic strategy was also applied to encapsulate vitamin A and E. The microcapsules synthesized by ultrasound were shown to be stable and preserve nutrients activity when embedded in staple food. The stability of the microcapsules was assessed by exposing them to simulated storage/transit conditions, including temperature (40 °C), moisture and UV irradiation. In the *in-vitro* digestion model, the biofunctionality and interfacial/microstructure characterizations were conducted to study the release process of encapsulated vitamin D and compared it with that of free vitamin D.

## 2 Material and methods

### 2.1 Chemicals and materials

Egg whites were obtained from hen eggs purchased from a local market and separating the natural egg yolk. Oral vitamin D, ferrous sulfate tablet, vitamin A and E from brands Ostelin®, Fero-grad® and Blackmore®, respectively were purchased from a local pharmacy. Commercial Twinings® green tea bag and plain flour were purchased from a local market. Tris (hydroxymethyl) aminomethane, hydrochloric acid (HCl), rhodamine B, nile red, mucin from porcine stomach, pepsin from porcine gastric mucosa, bile salts methanol and acetone were purchased from Sigma-Aldrich. High purity water with typical resistivity of 18.2 MΩ·cm at 25 °C was obtained from an inline Millipore RiOs/Origin system (Milli-Q water). The egg white solution (5% w/v) was prepared in Tris-HCl buffer (50 mM, pH 8.3). Then the 5% egg white solution was centrifuged, and the clear supernatant was collected for further use.

### 2.2 Formulation of vitamin filled microcapsules

**Fig. 1:**
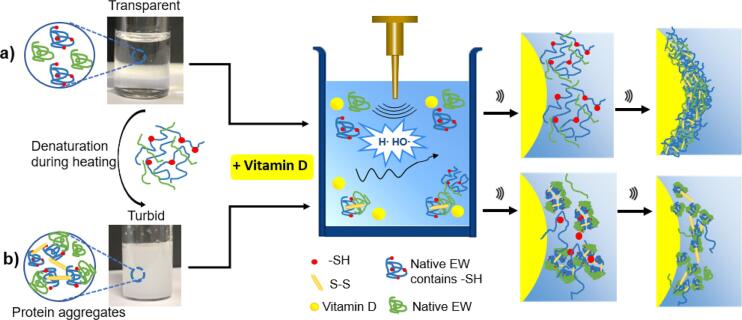
Schematic diagram of egg white microcapsules formation processes using native egg white solution (a) and thermal denatured egg white solution (b), respectively.

A 20 kHz ultrasound (Branson Digital Sonofier) generator with a standard titanium horn tip of 3 mm diameter was employed for synthesizing microcapsules (Cavalieri et al., 2008, Ye et al., 2016, Zhou et al., 2010). Vitamin D filled egg white shelled microcapsules were obtained by layering vitamin D (50 µL) on the surface of aqueous egg white solution (1 mL) and then sonicating at 160 W for 1 min by placing the horn tip at the oil/aqueous (vitamin D/egg white solution) interface (SI-Fig. 1). The microcapsules were separated from the remaining protein solution by flotation and copious washing with Milli-Q water by repeating the process (separation and washing) a few times (Cavalieri et al., 2008). Vitamin A and E filled microcapsules were both synthesized using the same method as mentioned.

Green tea (GT) infusions were prepared by submerging two tea bags in 200 mL hot (~80 °C) water for 5 min, cooled to room temperature (~25 °C), and filtered through a 0.22 µm membrane filter (Rahim et al., 2018). Iron solution (10 mg/mL) was obtained by dissolving one ferrous sulfate tablet in 10 mL milli-Q water, filtered through 0.22 µm membrane filters to remove insoluble impurities. Subsequently, the GT infusions and the iron solution were mixed (ratio 4:1) under a vortex mixer for 10 s. The mixed solution was centrifuged at 855*g* for 1 min (Megafuge™ 8 benchtop centrifuge), and then the dark purple supernatant was collected. The collected green tea/iron (GT/Fe^II/III^) complex solution (200 µL) was then added to the microcapsule’s suspension (1 mL) under a constant orbital shaker (40 rpm) for 2 min (Rahim et al., 2018). The GT/Fe^II/III^ coated microcapsules were obtained by discarding the excess supernatant after centrifugation (95*g*, 1 min), and resuspended in 500 µL of Milli-Q water. This procedure was repeated a few times to remove unreacted protein, tannic acid and iron in the solution. In order to confirm no contaminants remained in the suspension, the supernatant (following washing) was analyzed by UV spectroscopy.

### 2.3 Antimicrobial activity of microcapsules

The EW microcapsules were analyzed by a turbidimetric *Micrococcus luteus* test of enzymatic activity (Ibrahim et al., 1996). EW microcapsules (200 µL) was added to 3 mL of phosphate buffer containing 2 mg/ml *Micrococcus lysodeikticus*. The change in absorbance of the suspension at 450 nm was measured by a Cary 50 Bio UV-spectrophotometer for 6 min.

### 2.4 Microstructural characterization

Optical microscopy (Olympus Model IX71) was used to record the images of microcapsules dispersed in water and embedded into dough. The average size and size distribution of microcapsules were assessed by measuring over 300 microcapsules via optical microscopic images. Fluorescence properties of microcapsules (Rhodamine B dye egg white protein; Nile red dye vitamin D) were observed by using an inverted Olympus IX 71 wide-field fluorescence microscope with 60X objective lens. The morphology and thickness of the microcapsules were observed using a scanning electron microscope (FEI Teneo Volume Scope) at an acceleration voltage of 10 keV. The samples were mounted on a carbon-film copper grid from ProSciTech® and loaded with a single tilt holder. The samples were air-dried and pre-treated under vacuum, and then sputter-coated with a thin gold film.

The zeta potential of microcapsules/oil droplets before and after digestion were analyzed by Dynamic Light Scattering (DLS) with a Zetasizer Nano ZS from Malvern Instruments Ltd. Zeta potential measurements were carried out in disposable folded capillary cells, after equilibration for 30 s at 25 °C. The pH value of microcapsules after exposure to different digestion phases was measured by benchtop pH meter at room temperature.

### 2.5 Microcapsules incorporated in bread making processes

Dough made with plain flour and water was used as an example food matrix. A suspension of vitamin d-loaded egg white microcapsules (vitamin D solution contained fluorescent Nile red dye for imaging purpose) was added into the plain flour (ratio 3: 5) by following a kneading operation via 10-min hand or 2-min KitchenAid® machine. After this, a small piece of dough (microcapsules and flour mixture) was taken to be used as a thin slice for observing the microcapsules structures in the dough. For comparison, the same amount of Nile red labelled Vitamin D emulsion (free Vitamin D) was directly added into the dough, that subsequently went through kneading processes. The encapsulated/free vitamin D embedded into the dough went through dry heating (15 min 220 °C) in an oven. This heating procedure simulates the biscuit/bread baking process. After baking, the cooked dough was re-dispersed in methanol by using a blender (Breville®) for 5 min. After centrifugation (2375*g*, 5 min), the solid matrices were discarded, and the supernatant was filtered by 0.22 filter membrane for further HPLC analysis. Besides, to assess the actual temperature of microcapsules during the baking process, we then carried out the internal temperature measurement of the bread at different position (SI-Fig. 4, top and middle layer) by inserting a thermometer in the bread at different time (Channaiah et al., 2017).

### 2.6 In-vitro digestion model

The vitamin D filled microcapsules were passed through a simulated gastrointestinal tract (GIT) that included mouth, stomach and small intestine phases (Gu et al., 2017, Zhang et al., 2016). Vitamin D emulsion (free Vitamin D) as a control sample was also studied under the same GIT conditions. The microcapsules and simulated saliva fluid were preheated to 37 °C by water bath at a rotation speed of 100 rpm. First, the microcapsules and simulated saliva fluid (3 mL:3 mL) containing 0.03 g/mL mucin was adjusted to pH 6.8. Then,the digestion process started by incubating the initial mixture for 2 min at 37 °C. Simulated gastric fluid (6 mL) containing 0.2 % w/v pepsin was added into the initial mixture, and the value of pH was adjusted to 2.5. Subsequently, the mixture was incubated for 2 h at 37 °C to mimic stomach conditions. Finally, 3 mL simulated intestine fluid (containing 0.2% w/v pancreatin and 1.2% w/v bovine bile) was added to the mixture solution from stomach and incubated for 2 h at 37 °C, pH maintaining at 7.0. The composition of the simulated GIT fluid used is listed in (SI-Table 1).

### 2.7 Determination of vitamin recovery

Vitamin D microcapsules and emulsion were suspended in water and then heated at 40 °C for 20 h in water bath to determine the stability of vitamin D (Anselmo et al., 2019). The samples were irradiated by UV light (22 V, 700 mA) for 8 h to study the protection of egg whites from vitamin D degradation (Anselmo et al., 2019). To mimic the cooking condition, microcapsules and emulsion were embedded into a flour dough for baking at 220 °C for 15 min (Bredariol, Spatti, & Vanin, 2019). After the treatment (water bath heating, UV irradiation and baking), samples were collected for nutrients recovery determination. Microcapsule samples were broken down using high intensity ultrasound (120 W) for 30 s. Then methanol (ratio to sample 2:1) was used to dissolve Vitamin D, followed by centrifugation and filtration to remove the egg white shell (0.22 µm filter). Shimadzu SCL-10AVP high-performance liquid chromatography (HPLC) equipped with a LC-10 AT pump, liquid chromatography with a RESTEK column model Ultra AQ C18 5 µm (150 × 4.6 mm) and a UV/Vis detector (260 nm) was used to perform HPLC analysis (Anselmo et al., 2019). Samples were injected in volumes of 20 µL by SCL-10 AVP injector (flow rate 0.4 mL/min). HPLC analyses of vitamin A and E recovery was performed using acetone as an eluent at 25 °C with UV detector set at 325 nm following a similar method mentioned in vitamin D recovery experiments.

### 2.8 Statistical analysis

The data was statically analyzed with Minitab 19 software. Statistical analysis was performed using one-way analysis of variance (ANOVA) to identify which groups showed a statistically significant difference from others. The least significant difference values were set at the 5% level.

## 3 Results and discussion

### 3.1 Formulation of vitamin filled egg white microcapsules

Microcapsules were obtained by ultrasonic emulsification of vitamin D and EW diluted in Tris buffer (Fig. 1a). During sonication (20 kHz, 160 W, 1 min), the EW proteins stabilized the vitamin D droplets by adsorbing at the oil–water interface. The adsorption and crosslinking are promoted by the acoustic cavitation process, which is also responsible for the emulsification process (Lee et al., 2019). The average size of vitamin D filled microcapsules was 5.2 ± 1.6 µm. In our previous work, we have noted that lysozyme and albumin need to be firstly denatured before applying sound waves (ultrasound) in order to expose hydrophobic domains and free thiol groups to form stable microbubbles/capsules (Cavalieri et al., 2008, Cui et al., 2013). In the current study, to avoid external toxic chemical reducing agents, egg white (EW) suspension was treated by heating (5 min, 90 °C) to thermally denature the proteins (Fig. 1b). For comparison, untreated egg white was also emulsified by ultrasound (Fig. 1a) to evaluate the difference in morphology and stability of the obtained microcapsules.

**Fig. 2:**
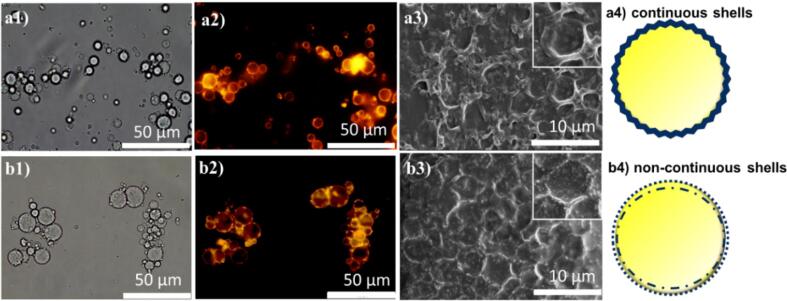
Optical, fluorescence, scanning electronic microscopic images and shell structure of microcapsules synthesized with unheated (a1,2,3,4) and heated egg white solution (b1,2,3,4), respectively. Rhodamine B was used to dye the EW protein in solution; SEM insets zoom in the microcapsules shell.

**Fig. 3:**
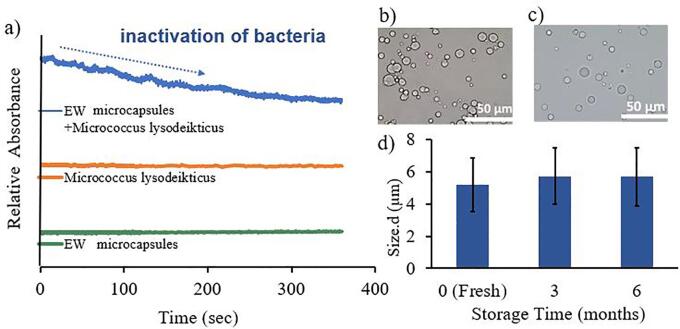
a) Enzymatic activity of EW microcapsules against *Micrococcus lysodeikticus* bacteria (blue curve); enzymatic activity in the absence of the EW microcapsules (orange curve) and only microcapsules suspension (green curve) (Lee et al., 2019); b) and c) optical microscopy imaging of EW microcapsules after 3 and 6 months storage; d) size of microcapsules as a function of storage time. (For interpretation of the references to colour in this figure legend, the reader is referred to the web version of this article.)

Optical/fluorescence and electron microscopic characterization provided further insight into the formation mechanism of microcapsules. As shown in Fig. 2a1, b1, both unheated and heated EW solutions can form microcapsules. The fluorescence images also show the composite structure of the EW microcapsules shell (Fig. 2a2, b2), where Rhodamine B was used to label the EW protein in the aqueous phase. Small protein particles on the surface are visualized in the heated samples (Fig. 2b2), whereas the shell of unheated samples is less segmented. The SEM images (Fig. 2a3, b3) show thick deflated microcapsules after they went through high vacuum process during the acquisition process. The thicknesses of the unheated and heated EW microcapsules’ shell were 0.21 ± 0.08 µm and 0.18 ± 0.06 µm, respectively, measured using SEM images (Ye et al., 2018). The mechanical properties of two types of microcapsules were determined by force-displacement curves using AFM (SI-Fig. 2) indicating unheated samples (185.1 ± 54.5 mN/m) possess stronger shell stiffness than heated samples (68.4 ± 16.2 mN/m). Thermal stability testing showed that unheated samples are more thermostable than heated samples. After heating in a 95 °C water bath for 1 h, the microcapsules made using heated egg white solution had almost disappeared (SI-Fig. 3), while the microcapsules made with unheated egg white solution were still intact. The observed difference in mechanical property and thermal stability could be ascribed to the morphology of the microcapsules’ shells (Fig. 1a4 and b4). The inset SEM images of the two samples (Fig. 1a3 and b3) showed the morphology of the unheated sample surface is found to be smooth, whereas small nanoparticles (dots) on the shell surface of the heated samples were noticed.

These nanoparticles are likely formed by the thermal denaturation and then aggregation of proteins. In the heated EW solution, the weak intramolecular interactions are easily broken, which causes EW protein unfolding and aggregating. The aggregation is mediated by intermolecular disulphide bonds, (Fig. 1b). After applying ultrasound, the remaining soluble proteins and small aggregates adsorbed at the oil (vitamin D droplets)-water interface and formed a composite shell likely stabilized by hydrophobic interactions and intermolecular-disulphide linkages. The morphology of the shell is segmented and non-continuous, which may result in thermal instability of microcapsules.

In the unheated sample (Fig. 1a), native EW proteins can be directly denatured at the oil–water interface during the ultrasonic emulsification process. This is due to ovalbumin being easily unfolded by shear forces, and ovotransferrin/lysozyme being sensitive to thermal denaturation due to the local heating induced by cavitation bubble collapse after applying ultrasound (Desert et al., 2001, Van der Plancken et al., 2005). These ultrasonically denatured proteins containing sufficient free thiol groups can be adsorbed and crosslinked at the vitamin D droplet interface, resulting in a continuous and stable shell stabilized by hydrophobic interactions and disulfide bond linkages. The surface tension measurements (EW- vitamin D) also showed that the native EW solution possesses better surfactant properties (20.10 ± 0.28 mN/m) for vitamin D than pre-heated EW (26.85 ± 1.06 mN/m). This observation indicates that native EW proteins better adsorbed at the oil-droplets interface to form a homogenous and more stable shells. Overall, this result suggests that the availability of free thiol group in solution is crucial to form a robust shell during microcapsules fabrication process.

### 3.2 Antimicrobial activity and shelf-life

Lysozyme from egg white has been reported to act as an antibacterial protein, which is known to degrade the peptidoglycan layer in the cell walls of Gram-positive bacteria by hydrolyzing the bond between *N*-acetyl muramic acid and *N*-acetyl glucosamine (Ibrahim et al., 1996, Masschalck and Michiels, 2003). The turbidimetric assay was performed to determine lysozyme activity using dead cells of *Micrococcus lysodeikticus* as substrate (Cavalieri et al., 2008, Lee et al., 2019). A decrease in the turbidity as a function of time was observed when the mixture of bacteria and microcapsules was analyzed (Fig. 3), indicating the lysis of *Micrococcus lysodeikticus*. The reduction in the turbidity demonstrated the EW microcapsules still possess antimicrobial activity due to the presence of lysozyme. We also tested the microcapsules and bacteria suspensions as control samples, respectively to consider the floating of microcapsules to the top of the solution or the sedimentation of bacteria to the bottom. The turbidity of both samples did not show any changes during the observation time (6 min). Therefore, these results confirm that the degradation of bacteria was induced by antibacterial function of EW microcapsules.

The integrity and shelf-life of microcapsules were evaluated by measuring the size of EW microcapsules as a function of time. The EW microcapsules maintained their structures well after 3 months and 6 months storage (Fig. 3b, c). The size of fresh, 3 months and 6 months stored microcapsules were 5 ± 2 μm, 6 ± 2 μm, and 6 ± 2 μm, respectively, (Fig. 3d). The high colloidal stability combined with the antibacterial property of EW microcapsules potentially allow EW microcapsules to have long-shelf lives during transit and storage processes.

**Fig. 4:**
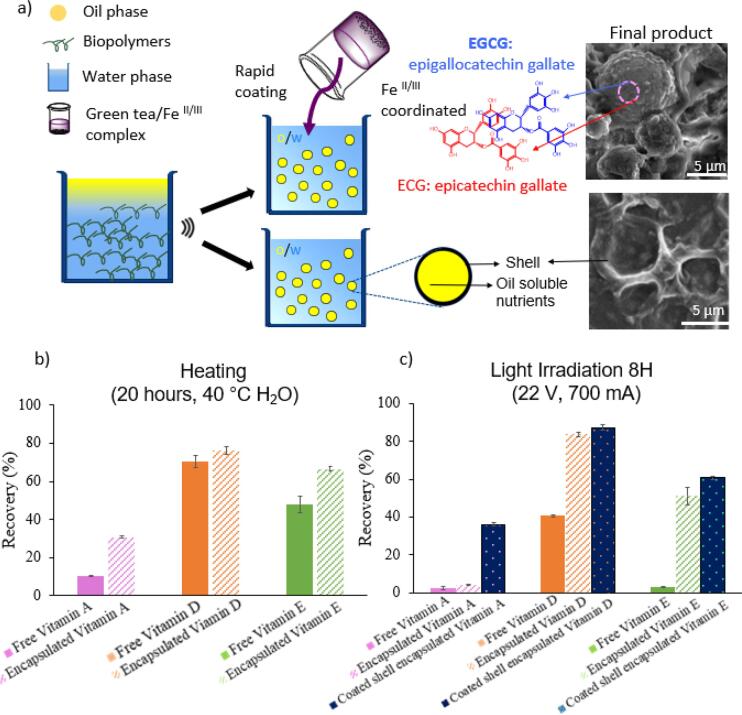
a) Schematic representation of the processes for formulating oil-soluble microcapsules and GT/Fe^II/III^ microcapsules; b) & c) recovery of encapsulated (or GT/Fe^II/III^ coated) versus unencapsulated nutrients after exposure to 40 °C heating water and UV light.

Apart from Vitamin D filled microcapsules, other lipophilic (oil-soluble) micronutrients such vitamin A and vitamin E have also been successfully encapsulated into egg white shells using the same ultrasonic method (Fig. 4a). The size of vitamin A and vitamin E filled microcapsules is 5.3 ± 1.3 µm and 5.3 ± 1.2 µm, respectively. There is no significant difference in size between these three oil-soluble nutrients formulated microcapsules (*p > 0.05) and all of them possess long-shelf life. Overall, these results indicate the high versatility and feasibility of the ultrasonic approach to synthesize microcapsules using raw hen egg whites.

### 3.3 Stability and functionality testing of microcapsules

Many nutrients are sensitive to temperature, moisture and ultraviolet light, which can cause degradation and thus limited absorption after ingestion. Although the high colloidal stability allows EW microcapsules to maintain their structure well up to 6 months, the role of microcapsules in improving encapsulated micronutrients stability against detrimental effects (such as high temperature, moisture, UV irradiation, etc.) is vital; hence required a comprehensive investigation. To improve the ability of UV absorption, we developed an additional coating method using green tea/iron complex on top of the egg whites’ shells (Fig. 4a).

We first investigated the preservation of the nutrients payload after 20 h heating in 40 °C water bath for both encapsulated and free micronutrients. This mimicked the transit environment under high temperature and moisture conditions. For the encapsulated micronutrients, more than 20%, 6% and 18% enhanced recovery was observed in vitamin A, D and E, respectively, as compared to non-encapsulated counterparts (Fig. 4b). We next investigated the preservation of micronutrients payload after 8 h UV light exposure. It was found that free vitamin A and vitamin E were significantly degraded, and 60% of vitamin D was degraded by UV light irradiation (Fig. 4c). Although we observed that the encapsulated vitamin D and E have more than 2-fold and 11-fold recovery than the non-encapsulated samples, there is no significant recovery improvement for Vitamin A when encapsulated. This result shows that the egg white shells can block/filter the UV light to some extent for protecting nutrients degradation, however the single protein shell has limited efficacy in protecting highly light-sensitive nutrients like vitamin A.

It is noticed that natural phenolic molecules have the ability to strongly absorb UV light (Zayat, Garcia-Parejo, & Levy, 2007). Green tea (GT) has been reported as a common and rich source of phenolic compounds, containing epigallocatechin gallate (EGCG), epicatechin gallate (ECG). GT can form metal-phenolic networks by coordination-driven assembly (Rahim et al., 2018). We developed an additional coating method using green tea/iron complex to produce microcapsules that can protect the micronutrients from ultraviolet (UV) radiation. This food grade phenol-metal complex (GT (EGCG/ECG)/Fe^II/III^) made from green tea and iron supplement was coated on the surface of egg-white shells as an external protective material for blocking/filtering UV light. Compared with the non-coated microcapsules, the green tea coated microcapsules presented a shell which did not collapse under SEM high vacuum condition (Fig. 4a). These results suggest that the green tea/Fe^II/III^ complex was adsorbed on the egg white shell surface, likely resulting in a stiffer and thicker double-layer protective shell encapsulating the nutrients. By using this additional coating process, the nutrients recovery upon UV light irradiation was significantly improved by more than 8-fold, 2-fold, 15-fold for vitamin A, D and E, respectively in the coated encapsulation samples, as compared to the non-encapsulated samples (Fig. 4c). Overall, the external green tea/Fe^II/III^ coating of microcapsules significantly improve the stability of the encapsulated nutrients against light exposure and combine vitamins with iron supplements. Compared with free vitamin counterparts, encapsulated vitamins were well protected from degradation under heating, moisture, UV irradiation due to the egg white shells protection.

**Fig. 5:**
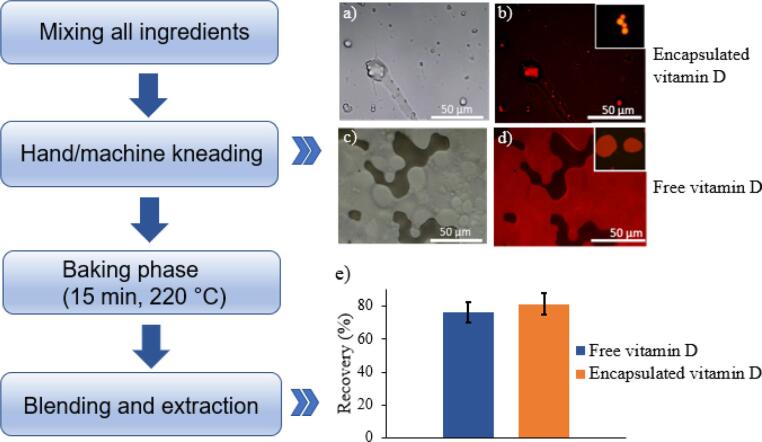
The process of incorporating microcapsules into dough. a), b) optical and fluorescent imaging of the vitamin D (dyed with Nile red) microcapsules embedded into dough by hand kneading; c), d) optical and fluorescent imaging of free vitamin D embedded into dough by hand kneading, fluorescent imaging insets present sample microstructures under machine kneading (scale bar is 50 µm); e) recovery of encapsulated vitamin D and free vitamin D, which extracted from the cooked dough. (For interpretation of the references to colour in this figure legend, the reader is referred to the web version of this article.)

We next evaluated the incorporation of vitamin D loaded microcapsules into common food products and their stability under cooking conditions. For instance, baking processes have typically deleterious effects on vitamins stability. A dough made with plain flour and water was used as a model food matrix. Vitamin D loaded microcapsules were embedded into the dough. We first verified if the microcapsules embedded into the dough can maintain their structure during typical dough kneading process that imparts compressive mechanical stresses on the microcapsules. Optical and fluorescence microscopy images (Fig. 5a and b) clearly show that the EW microcapsules embedded into the processed dough still maintained their structures without bursting and releasing the nutrients into the food matrix. The red dots indicate that the oily phase (vitamin D) labelled with Nile red did not leak out. For comparison, the same amount of non-encapsulated vitamin D dyed with Nile red was directly added into the dough (Fig. 5c and d). We observed that the vitamin D droplets in the absence of EW shell protection, merged together to form large oily regions in the dough because of the coalescence of vitamin D. This result demonstrates that EW microcapsules are resistant to mechanical stresses and maintain their structure during kneading processes.

Next, the thermal functionality test of the encapsulated vitamin D was performed. The microcapsules embedded into to the dough went through dry heating (15 min 220 °C) in an oven. This heating procedure simulates the biscuit/bread baking process. After cooking, bread fortified with microcapsules showed a higher vitamin D recovery (81.3% ± 6.3%) than that with free vitamin D, indicating that the microcapsules embedded into food matrices are thermostable and that the vitamin D was protected from degradation to a certain extent. In this specific cooking process, the maximum internal temperature is below 100 °C (SI-Fig. 4). As it is known, the food matrices internal temperature is usually much lower than the oven ambient temperature (Channaiah et al., 2017, Dumas and Mittal, 2002). Many other food products can even be cooked at less than 220 °C and in less time, such as cookies, and roti (Indian Flat Bread), etc. Therefore, the prospect of vitamin D filled microcapsules embedded into food products to improve the nutritional quality is highly promising. In addition, a film of Vitamin D loaded capsules can be sprayed on top of the dough after cooking during the cooling process, to prevent the thermal degradation of Vitamin D.

### 3.4 Influence of GIT conditions on vitamin D bioavailability

It has been demonstrated that the shell of EW microcapsules is highly stable and can protect micronutrients payloads during exposure to simulated transit/cooking processes. In terms of improving the nutritional value of common food, the stability and absorption of the nutrients after ingestion in human body is crucial. Hence, we prepared a simulated gastrointestinal tract (GIT) model for nutrients absorption assessment, which consists of mouth (pH~6.8), stomach (pH~2.5), and small intestine phases (pH~7) (Gu et al., 2017). Three delivery systems, free vitamin D (emulsion), encapsulated vitamin D (microcapsules), and coated shell encapsulated vitamin D (GT/Fe^II/III^ coated microcapsules) were incubated with the three different media (simulated saliva, simulated gastric fluid and simulated intestine fluid) for comparative study.

**Fig. 6:**
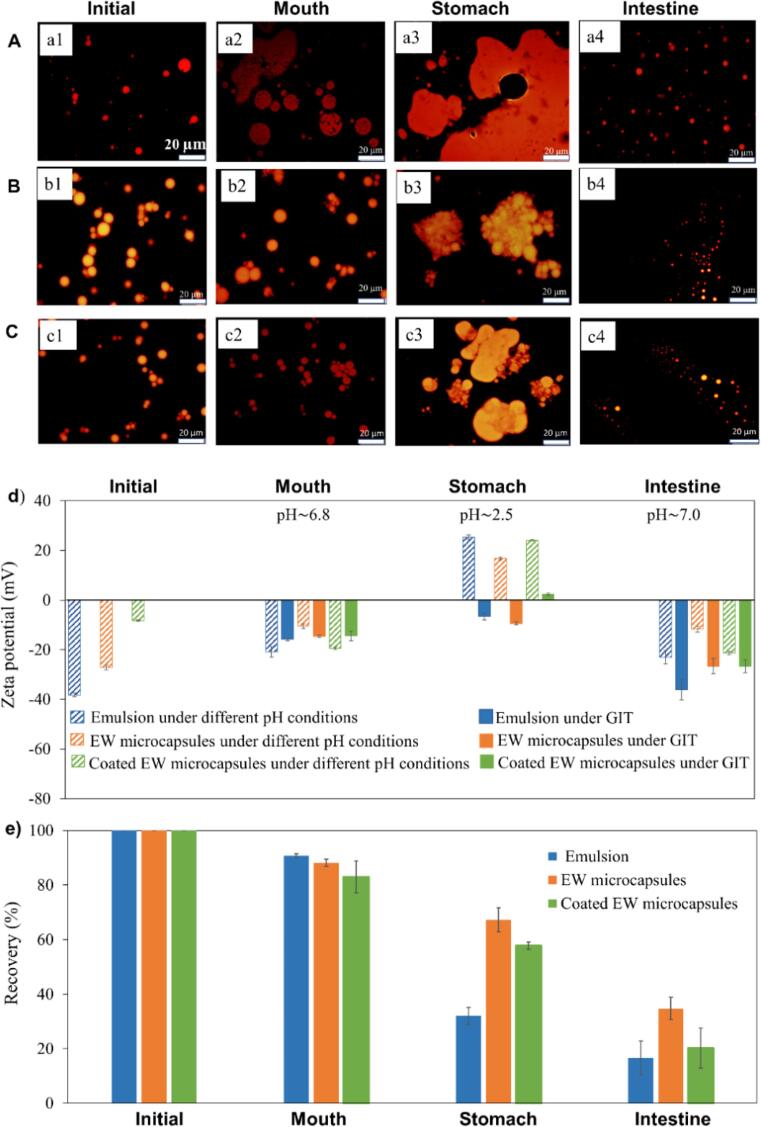
Characterization of different delivery systems: emulsion, microcapsules, GT/Fe II/III coated microcapsules, respectively. A), B), C) fluorescence images of different Vitamin D delivery systems after exposure to the simulated GIT. A), B) and C) present free vitamin D (emulsion), encapsulated Vitamin D (microcapsules), and coated shell encapsulated vitamin D (GT/Fe II/III microcapsules) system, respectively (scale bar is 20 µm); d) electrical characteristics (ζ potential) of three delivery systems under different pH conditions and after exposure to the simulated GIT stages; e) nutrients recovery of three delivery systems determined using HPLC in in-vitro digestion model.

The three different systems initially showed relatively small mean particle size (Fig. 6a1, b1, c1). After they were exposed to the simulated mouth medium, the size of the uncoated emulsion increased considerably, and droplets coalescence occurred (Fig. 6a2). However, both microcapsules and coated microcapsules still maintained their size and structure very well (Fig. 6b2, c2). After they were exposed to the stomach medium, the uncoated emulsion was completely broken and separated into a non-dispersed oil phase (Fig. 6a3). Vitamin D is known to isomerise and/or degrade under various conditions, including the acid conditions encountered in the stomach. Conversely both microcapsules and coated microcapsules partially maintained their integrity, forming aggregated clusters (Fig. 6b3, c3). Of note in the stomach medium, the egg white proteins can be digested by hydrolytic enzymes (pepsin), that caused the exfoliation of protective protein shells, resulting in big clusters. The bigger clusters caused the brighter fluorescence imagines due to dye (Nile red) accumulation. The results of sample morphology change in the stomach phase indicate that the EW shell of the microcapsules and coated microcapsules can be slowly degraded resulting in a sustained release of nutrients.

Interestingly, after exposing the three systems to the small intestinal medium, we found that both the bulk oil phase (Fig. 6a3) and the particles aggregates (Fig. 6b3, c3) become small droplets in all of the three samples (Fig. 6a4, b4, c4). This observation could be ascribed to that the pancreatin and bile salt in the intestinal fluid typically broke down vitamin D (bulk oil phase and aggregates) and hydroxylated lipids, resulting in smaller droplets. (Nair & Maseeh, 2012). Overall, our results indicate that the EW and GT/Fe^II/III^ coated EW shells of microcapsules prevent the rapid coalescence of vitamin D in a bulk oil phase occurring in the stomach. Both microcapsules eventually disassemble and release vitamin D in the intestine.

The interfacial properties of emulsions, microcapsules and GT/Fe ^II/III^ coated microcapsules are other aspects that need to be studied because they are related to the changes in the particle size/microstructures when they were exposed to different media. Therefore, zeta potential (ζ potential) measurements were carried out. We first investigated the effect of pH on zeta potential for those three delivery systems. All systems are expected to carry a negative charge below pH 5, a positive charge above pH 5 and neutral at pH 5 (SI-Fig. 5). This is explained by the isoelectric points of the proteins constituting the microcapsules shell. In the GIT digestion model, it was observed that the initial emulsions and uncoated microcapsules (initial pH~8.3) had high negative surface potential (Fig. 6d) which confers the systems colloidal stability as a result of the strong electrostatic repulsion. Coated microcapsules showed less negative surface charge due to the GT/Fe^II/III^ coating on top of the protein shell (initial pH~6.0), which is likely induced by the positive metal ions. After passing the mouth medium at pH to 6.8, all samples showed less negative surface potentials (Fig. 6d). However, as they passed through the stomach phase, emulsion and microcapsules still showed negative charge and we noted that the emulsion droplets were broken, and aggregation occurred (Fig. 6a3, b3, c3). This phenomenon could be ascribed to the adsorption of some anionic species (mucin) adsorbed to the surfaces of the lipid droplet (emulsion) and protein coated lipid droplets (microcapsules). The zeta potential of the vitamin D droplets formed in the intestine medium was strongly negative, which might be due to the adsorption of residues of surface-active anionic species (peptides, bile salts, free fatty acid) to the droplets surface.

The ability of the three systems to deliver functional vitamin D was investigated while passing the various stages of GIT mode (Fig. 6d). There is no significant change of vitamin D recovery for all samples after passing through mouth phase. After they were exposed to the stomach phase, the emulsion sample showed a dramatic decrease of vitamin D recovery, while microcapsule samples showed much less decrease in nutrient recovery. It is known that the acidic pH of gastric fluid may affect the bioavailability of vitamin D (Maurya & Aggarwal, 2017). For the uncoated emulsion sample, the free vitamin D could be directly degraded into a bio-inactive form in the acidic gastric juice. Due to the presence of pepsin, the protective egg white shell could be digested and exfoliated, the encapsulated vitamin D droplet would be exposed to the acidic degradation as well. The nutrients recovery for coated microcapsules and non-coated samples are similar because the GT/Fe^II/III^ coating is likely easily disassembled in acidic condition. Both the acidic pH of stomach fluid (~pH 2.5) and the presence of pepsin (powerful digestive enzyme) are responsible for the less vitamin D recovery in the stomach phase.

As the intestine phase is responsible for vitamin D absorption, these results suggest that microcapsules enable more than 67% of vitamin D to reach the intestine in the active form for absorption, while the uncoated emulsion allows only 32% of ingested vitamin to be absorbed in intestine because of the degradation in stomach (Fig. 6d). The coated microcapsules have a similar decrease in the rate of nutrients recovery indicating that the GT/Fe^II/III^ coating did not affect the nutrient's availability. In the intestine phase, the presence of pancreatin and bile salts are responsible for the less nutrient recovery because they can break down vitamin D droplets and hydroxylate lipids. The bile salts generated from liver can cause vitamin D to be hydroxylated into 25-hydroxyvitamin D (25 [OH]D) (Nair et al., 2012). In this case, less vitamin D recovery was observed in the intestine phase due to the hydroxylation. Overall, ultrasound-assisted microencapsulation can potentially improve vitamins absorption more than 2-fold. Considering the loss of free nutrients during storage, transit and cooking processes, these microcapsules provide much more promising opportunities to improve food nutritional value by embedding them into common products.

## 4 Conclusions

The ultrasound-assisted formation of oil-soluble vitamin (A, D and E) filled microcapsules from raw hen egg whites has been successfully achieved. It is noted that the high availability of free thiol groups in protein solution is crucial to form stable microcapsules with robust shells. The thermal stability and nutrients functionality testing demonstrated that the microcapsules could be embedded into food products while maintaining the nutrients functionality. The long-shelf microcapsules can protect micronutrients payloads during exposure to high temperature, moisture, UV light irradiation, mechanical stress and enable slow release of nutrients after ingestion. Such highly stable micronutrient filled microcapsules are promising to be applied in food industry to enhance nutritional value of staple foods.

## CRediT authorship contribution statement

**Haiyan Zhu:** Conceptualization, Investigation, Visualization, Writing - original draft. **Srinivas Mettu:** Conceptualization, Funding acquisition, Methodology, Investigation, Writing - review & editing. **Francesca Cavalieri:** Conceptualization, Funding acquisition, Supervision, Methodology, Writing - review & editing. **Muthupandian Ashokkumar:** Conceptualization, Funding acquisition, Project administration, Supervision, Methodology, Writing - review & editing.

## Declaration of Competing Interest

The authors declare that they have no known competing financial interests or personal relationships that could have appeared to influence the work reported in this paper.
