# Supplementary material for: Ultrasonic microencapsulation of oil-soluble vitamins by hen egg white and green tea for fortification of food
Source: Food Chem. 2021 Aug 15;353:129432. doi: 10.1016/j.foodchem.2021.129432 (PMC8164159; doi:10.1016/j.foodchem.2021.129432)
Supplement: Supplementary data 1 [file mmc1.docx]

Supplementary Information

**SI-Table 1. Chemical composition of simulated GIT fluids in vitro digestion model**

| Digestion phase | Chemicals | Concentration |
| --- | --- | --- |
| Mouth phase | Ammonium sulfate | 1.03 mM |
|  | Monopotassium phosphate | 2.34 mM |
|  | Potassium chloride | 1.35 mM |
|  | Porcine gastric mucin (type II) | 15 mg/ mL |
|  | Sodium chloride | 13.64 mM |
|  | Tri-sodium citrate | 0.5 mM |
|  | Urea | 1.65 mM |
|  | Uric acid (sodium salt) | 0.06 mM |
| Stomach phase | Sodium chloride | 34 mM |
|  | Hydrochloric acid | 50.00 mM |
|  | Pepsin from porcine gastric mucosa | 2.00 mg/ mL |
| Small intestine phase | Sodium chloride | 20.00 mM |
|  | Monopotassium phosphate | 50.00 mM |
|  | Bile salts | 12.00 mg/ mL |
|  | Pancreatin | 2.00 mg/ mL |


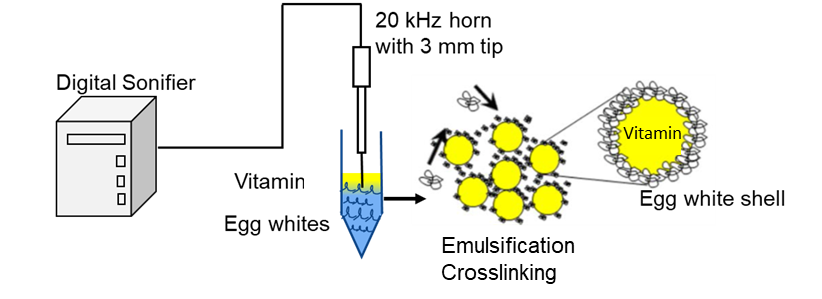


**SI-Figure 1. Schematic diagram of synthesis vitamin (A, D, E) filled egg white microcapsules**


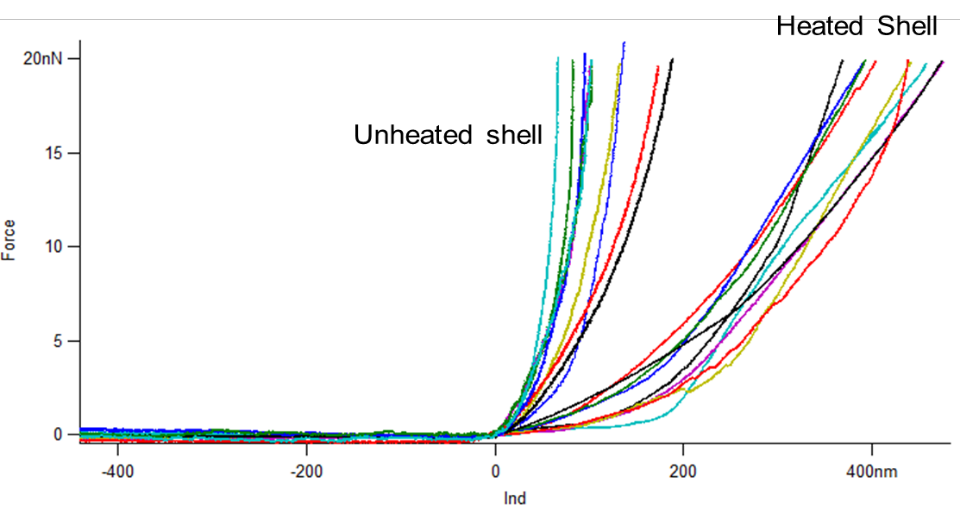


**SI-Figure 2. The analysed Force-indentation curves of the microcapsules prepared using unheated egg white solution and heated egg white solution. The slope of the force curves is proportional to the stiffness of the microsphere given the relation: *F=k_shell_ δ,* where *F* and *δ* are applied load and indentation respectively. *K_shell_* represents the microsphere stiffness.**


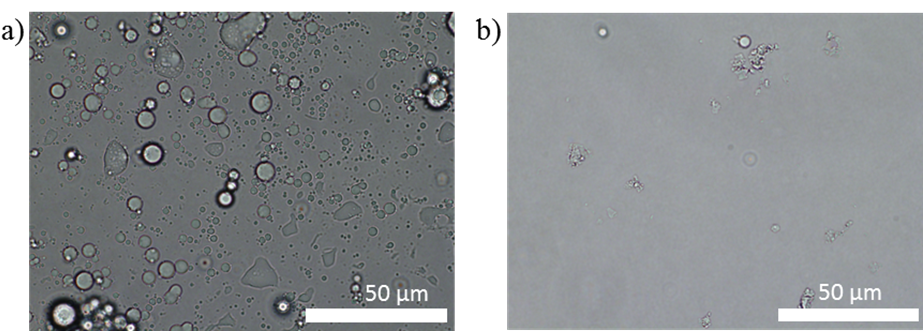


**SI-Figure 3: Thermal stability testing of microcapsules under water bath heating 95⁰C for 1 hour. a) microcapsules synthesized with non-heated egg white solution; b) microcapsules synthesized with pre-heated EW solution.**

**
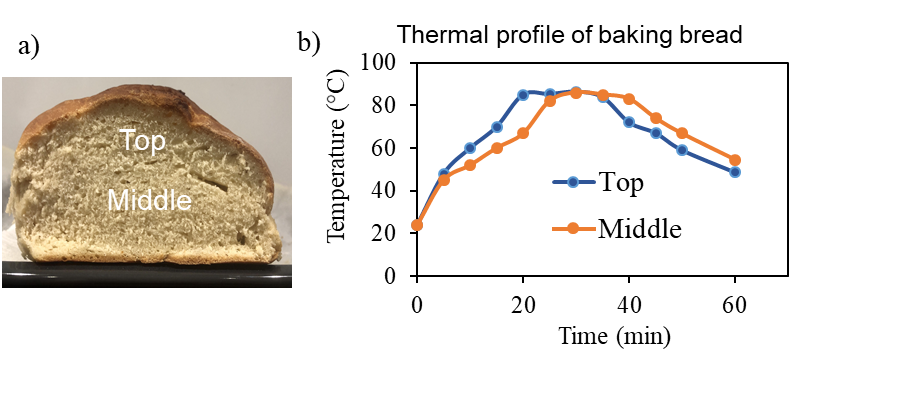
**

**SI-Figure 4: a) imaging of bread which baked for internal temperature measurement, the blue line pointed to the top layer of bread, the orange line pointed to the middle layer of the bread; b) mean internal temperature profile of bread during 30 min of baking at 220 °C oven temperature followed by 30 min of ambient cooling.**


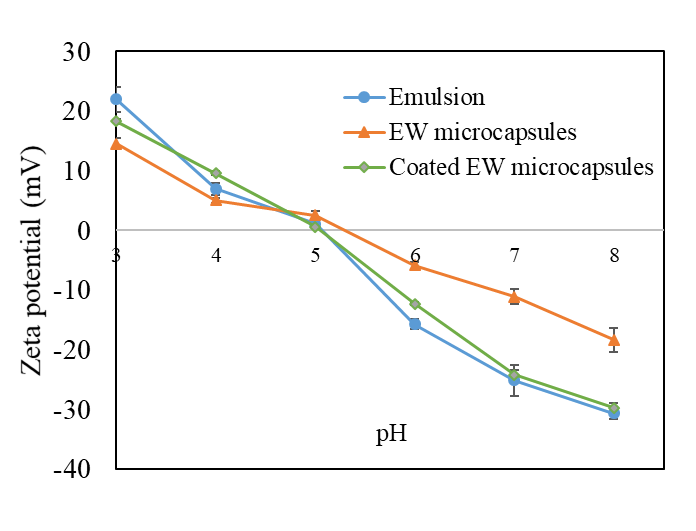


**SI-Figure 5. Influence of pH on the electrical characteristics (ζ potential) of different delivery systems under different pH conditions.**
